# Isothermal titration calorimetry of ion-coupled membrane transporters

**DOI:** 10.1016/j.ymeth.2015.01.012

**Published:** 2015-02-09

**Authors:** Olga Boudker, Oh SeCheol

**Affiliations:** Department of Physiology & Biophysics, Weill Cornell Medical College, New York, 10021 USA

## Abstract

Binding of ligands, ranging from proteins to ions, to membrane proteins is associated with absorption or release of heat that can be detected by isothermal titration calorimetry (ITC). Such measurements not only provide binding affinities but also afford direct access to thermodynamic parameters of binding - enthalpy, entropy and heat capacity. These parameters can be interpreted in a structural context, allow discrimination between different binding mechanisms and guide drug design. In this review, we introduce advantages and limitations of ITC as a methodology to study molecular interactions of membrane proteins. We further describe case studies where ITC was used to analyze thermodynamic linkage between ions and substrates in ion-coupled transporters. Similar type of linkage analysis will likely be applicable to a wide range of transporters, channels, and receptors.

## 1. Introduction

Majority of chemical reactions are associated with absorption or release of energy in the form of heat. Isothermal titration calorimetry (ITC) measures this heat, and thus the energy of a reaction directly as reactants are mixed in the instrument cell. Over the last several decades, ITC has been applied to numerous systems. Majority of these studies focused on molecular interactions, such as between proteins, or between proteins and small ligands, DNA or other macromolecular systems. Other phenomena, including enzyme kinetics have also been probed by ITC. Excellent reviews (1–4) are available covering appropriate methodologies including studies of membrane proteins (5). Though lagging behind soluble proteins, interactions of membrane proteins with a variety of partners are also being actively interrogated. An incomplete list includes channels binding ions (6–8) and gating ligands (9, 10), secondary transporters binding substrates and coupled ions (11–26), and assembly of protein complexes (27, 28). ITC is routinely used to establish functionality and substrate specificity of channels and transporters. However, other questions such as stoichiometry of binding (26) and ion-coupling mechanisms (12, 15) have also been probed using this technique. Here, we will discuss applications of ITC to studies of membrane proteins with further focus on the mechanistic studies of ion-coupled transporters.

### 1.1. Advantages of ITC

There are two key advantages of ITC in studies of molecular interactions. First, ITC is performed on native proteins without a need for modifications. By contrast, approaches based on fluorescence spectroscopy require that at least one of the reactants is either intrinsically fluorescent or chemically labeled. Furthermore, reactants in ITC are in solution, as opposed to, for example, surface plasmon resonance spectroscopy, where one of the interacting components has to be surface-immobilized. Hence arguably, binding observed by ITC approximates binding processes in cells most closely among common in vitro methods. Finally, ITC is unaffected by spectroscopic properties of reactants. For example, there are no limitations on their internal fluorescence or optical density. This property allows one to study binding in solutions with variable compositions. In the case of membrane proteins, these include detergent micelles, bicelles or membrane mimetics, such as nanodiscs and proteo-liposomes. Importantly, ITC experiments are technically simple, requiring only basic training, and the instruments are widely available and relatively inexpensive.

Second, ITC provides rich thermodynamic information, including values of enthalpy (Δ*H*), entropy (Δ*S*) and heat capacity (Δ*C_p_*) of binding in addition to standard free energy (Δ*G°*) and dissociation constants (*K_D_*). Moreover, information on a reaction mechanism can often be obtained such as the number of binding sites (*n*), presence or absence of cooperativity, and coupling of ligand binding to protonation/deprotonation events. Access to thermodynamic parameters (Δ*H*, Δ*S* and Δ*C_p_*), which define the thermodynamic signature of a process, is of significant value in data interpretation. Collectively, these parameters determine the values of Δ*G°* and *K_D_* and their temperature dependencies.

(Eq. 1) $$K_{D}=\exp\left(-\frac{\Delta G{}^{\circ}}{RT}\right)$$

(Eq. 2) ΔG°=ΔH-TΔS,

(Eq. 3) $$\Delta H=\Delta H_{\mathrm{REF}}+\Delta C_{P}(T-T_{\mathrm{REF}})$$

(Eq. 4) $$\Delta S=\Delta S_{\mathrm{REF}}+\Delta C_{P}\ln\left(\frac{T}{T_{\mathrm{REF}}}\right),$$

where *R* is the gas constant, *T* is an absolute temperature, *T_REF_* is a reference temperature, and Δ*H_REF_* and Δ*S_REF_* are the reaction parameters at *T_REF_*. Importantly, Δ*G°* is the standard free energy defined as the free energy of binding at 1 M concentration of reactants. The relationships of Δ*H*, Δ*S* and Δ*C_p_* to structures and structural changes of interacting components have been extensively studied and challenges associated with these interpretations recognized (29, 30).

In general, Δ*C_p_* is probably the most information-rich parameter. It reflects the complexity of the underlying reaction inasmuch as the magnitude of Δ*C_p_* is related to the multiplicity of the formed cooperative weak interactions (31). Thus, binding of a small ligand to a preformed binding site is expected to produce small Δ*C_p_*. In contrast, reactions involving protein restructuring, rigidification and extensive changes of interactions with water are expected to produce large Δ*C_p_* (31–33). We discuss measurements and interpretation of Δ*C_p_* in **Section 4.3.**

Access to the thermodynamic signature of binding is of a special importance for drug development (34–37). Δ*H* is a direct measure of the collective energies of bonds that are made and broken during complex formation. These include ionic interactions, hydrogen bonds, and van der Waals interactions between protein and ligand, within the protein itself, and between interacting solutes and water. Hydrogen bonds formed between protein and ligand are the principal contributors to favorable Δ*H* of binding and are important for specificity of drug interactions with the target, as well as strength of binding. However, *in silico* modeling and optimization of the bonds is challenging because their strength depends on precise distance and orientation of relevant atoms. Therefore, selecting lead compounds that already show favorable binding Δ*H* for further optimization might be a useful strategy (34–37). Δ*S* of binding comprises cratic, solvation, and conformational entropies. While cratic ΔS is typically unfavorable, reflecting loss of rotational and translational degrees of freedom upon complex formation, solvation and conformational entropies can be both favorable and unfavorable. Solvation entropy is usually favorable and is due to dehydration of the hydrophobic regions of reactants and release of water molecules into the bulk. Conformational entropy is typically, but not always (38), unfavorable due to increased conformational constraints on both protein and ligand. Rational optimization of ΔS during drug maturation is relatively straightforward: addition of hydrophobic moieties to the drug will increase solvation entropy gains, and rigidifying the drug would decrease the conformational entropy penalty (29, 35).

### 1.2. Limitations of ITC

**Fig. 1:**
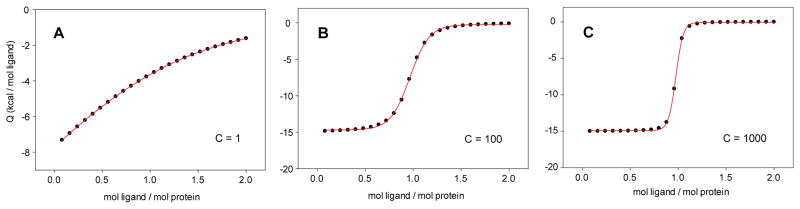
Binding isotherms for low (A) intermediate (B) and high (C) affinities Integrated injection heats produce binding isotherms, from which *K_D_*, Δ*H* and *n* are determined. Simulated data are shown with Δ*H* of −15 kcal/mol, *n* of 1. The solid red lines through the data are fits to the independent binding sites model. Corresponding *c* values are on the panels.

Ideally, protein concentration in ITC experiments should be between 10 and 500 fold over the *K_D_* of the complex. For a simple binding model of *n* identical independent binding sites, this requirement is usually discussed in terms of a parameter *c*:

(Eq. 5) $$c={nM}_{T}/K_{D},$$

where *M_T_* is the total protein concentration (39). Complexes that are too tight or too weak cannot be optimally studied (Fig. 1).

To stay within favorable range of *c* values for tight binding with *K_D_* below 1–10 nM, concentrations of the reactants may become too low to obtain good signal to noise levels. When *c* values exceed 1000, determination of binding *K_D_* becomes challenging (Fig 1A). However under high-*c* conditions, an upper bound of *K_D_* can always be obtained, and values of *n*, Δ*H* and Δ*C_p_* are very well determined. Notably, the sensitivity of ITC instruments is continuously improved and therefore with time, tighter complexes will become fully accessible. Improvements in hardware are accompanied by optimization of ITC data analysis procedures that now allow for more accurate baseline determination and error evaluation (40). Finally, if two competitive ligands are available with one binding too tightly, but the other binding within the suitable range, displacement titrations can yield all thermodynamic parameters for both ligands (41, 42). Typically, protein is first titrated with the moderate affinity ligand and then with the tight ligand, which displaces the weaker competitor. An optimized single titration protocol has been recently been described (43). From these experiments, the free energy and other parameters for the ligands are calculated based on competitive binding equilibrium equations.

Weak binding reactions with *K_D_* above ~100 μM also present problems (Fig 1B). For these reactions, very high protein and ligand concentrations are ideally required that may not be feasible to achieve. In experiments where *c* value is below ~2, Δ*H* and *n* are tightly correlated, but fairly accurate *K_D_*-s can still be obtained (42, 44). If binding stoichiometry *n* is known independently, then Δ*H* can also be determined after fixing *n* (see **Section 4.2** below for an example). In general, a priori knowledge or a hypothesis on the mechanism of binding will extend the range of useful data and allow application of more advanced data analysis programs, such as SEDPHAT (45). In addition, experimental strategies extending ITC applicability to the studies of weak binding are being developed (46, 47).

Difficulties may also arise from limited solubility or stability of either proteins or ligands. Many hydrophobic or amphipathic ligands would not be suitable for ITC. Such ligands may simply be insoluble at required concentrations, or they may form aggregates in solution. In the latter case, ligands may partition further into the solution during injections into the reaction cell. Such dissolution processes are often associated with significant heat effects that may obscure the heats of complex formation. In the case of membrane proteins, hydrophobic or amphipathic ligands may also partition into detergent micelles or other hydrophobic phases used to solubilize these proteins. Redistribution of ligands into such phases during titrations may again be associated with significant heat effects. Proteins themselves may not be sufficiently stable to remain in solution in native state throughout the duration of the experiment (~one hour) at appropriate temperature (typically 25 °C) and under extensive mechanical stirring.

Finally, it should be noted that even for excellent samples, experiments might not produce useful data if complex formation is not associated with significant heat effects. Most small ligands, which bind to proteins with the formation of at least a few hydrogen bonds, give Δ*H* values between −2 and −20 kcal/mol, which are sufficient for ITC measurements. However, enthalpy changes associated with conformational transitions in the protein in response to ligand binding may offset those due to complex formation yielding final enthalpy changes that are low. Because enthalpy is dependent on temperature (Eq. 3), performing experiments at a different temperature may often improve the signal.

## 2. Materials and methods

### 2.1. Protein sample preparation

Working with structurally stable homogeneous protein samples is critical during ITC. Protein unfolding and aggregation are associated with heat effects, and the presence of aggregates also contributes mechanical heat during stirring. All of these processes add noise or baseline drifts and fluctuations, obscuring signals from protein-ligand interactions. To prepare high-quality samples, we typically purify proteins by size exclusion chromatography (SEC) immediately prior to ITC experiments. Protein elution in a single narrow peak is a good (although not always sufficient) indication that the protein is in a native state. Notably, many proteins are greatly stabilized by ligands or substrates. Hence, we typically perform most of our protein isolation steps in the presence of ligands. These are removed during the final SEC step or by dialysis. Care should be taken when using tightly binding ligands with nanomolar affinities because they may not be efficiently removed by these procedures.

High protein purity is critical in ITC because contaminants result in errors during protein concentration determination. Such errors may lead to inaccurate determination of *K_D_* and Δ*H* (48). They also directly affect determination of ligand to protein binding stoichiometries, which becomes when these stoichiometries are questioned (26). Similarly, accurate measurements of protein concentration are also highly desirable. Typically, Edelhoch method is used, whereby light absorbance by protein solutions at 280 nm is determined (49). The absorbance is mostly due to the presence of tryptophans and, to a lesser extent, tyrosines and cystines (disulfide bonds). Protein concentration is calculated using Beer-Lambert law and molar extinction coefficient estimated from the protein amino acid sequence (50) (using, for example, PROTPARAM tool: http://web.expasy.org/protparam/(51)). To more accurately measure protein extinction coefficient and concentration, quantitative total amino acid analysis can be used (15, 26). This approach becomes particularly important when protein lacks tryptophans or when it contains optically active impurities or cofactors. Bradford assay (52), based on complex formation between Coomassie Blue dye and hydrophobic protein regions, is not recommended for membrane proteins because detergents interfere with dye binding to the protein and also directly interact with the dye.

### 2.2. Matching cell and syringe buffers

One important goal of ITC sample preparation is to match buffer compositions in the syringe and the cell so that the only difference between them is the presence of the protein and the ligand. If buffers are not identical, additional heats associated with the dilution of unmatched components will be observed and can be very large. For example, some ligands require buffers supplemented with dimethyl sulfoxide (DMSO) for solubility. This requirement is unfavorable for ITC because DMSO dilution heats are very large and may mask the reaction heats. Typically, proteins should be SEC-purified or extensively dialyzed against buffer identical to that used to solubilize the ligand to achieve a good match between cell and syringe solutions.

Literature review shows that a majority of ITC experiments have been conducted in n-decyl- and n-dodecyl-β,D-maltopyranosides and related detergents. Other detergents, – β-D-octylglucoside, Fos-Cholines and polyoxyethylene ethers – have also been used successfully (5). Matching detergent concentrations in the cell and the syringe is typically accomplished by preparing protein and ligand solutions in buffers containing identical concentrations of detergents. Occasionally, detergent matching has been achieved empirically by performing a series of dilution experiments (10). However, it is important to note that protein samples are usually concentrated prior to titration experiments. This process leads to concomitant concentration of detergent micelles. Therefore, the final concentration of detergent in the cell is unknown and most certainly higher than in the syringe. Nevertheless, we have not observed major problems related to detergent concentration mismatch, and, to our knowledge, such problems have not been reported. Detergent concentrations used in experiments are typically around 2–3 fold above the critical micelle concentration (CMC). Notably, CMC varies with pH, temperature, salt concentration, and in the presence of co-solvents (53). For example, DMSO in concentrations between 1 and 7.5 % increases CMC of β-D-octylglucoside about two-fold (54). CMCs tend to decrease with increased ionic strength (55, 56). Temperature dependencies differ for distinct detergents with CMCs typically increasing modestly (less than two-fold within the relevant temperature range) both at low and high temperatures (54, 56–58). While information on CMCs is available for many common detergents, there is also a multitude of methods to determine CMCs experimentally under the desired solution conditions, including simple calorimetric approaches (57–59). One case, in which we observed dilution artifacts, was using n-dodecyl-β,D-maltopyranoside at 2 x CMC concentration while performing titration experiments at temperatures above 40 °C. Doubling detergent concentrations readily resolved these artifacts.

### 2.3. Alternative hydrophobic phases

Membrane proteins can also be reconstituted and studied in bicelles (19, 60), nanodiscs (9, 28) or liposomes (18, 20, 61), which more closely approximate their physiologic environment. Such preparations may also allow investigations of the effects of varied lipid compositions on ligand binding. However at present, only a few ITC studies on membrane proteins have been performed in media other than detergent. Hence, it is difficult to judge how critical lipid environment will be for a “typical” protein. Studies comparing gating ligand cAMP binding to a potassium channel (9) and FhuA assembly with TonB (28) showed that binding thermodynamics were significantly different in detergent micelles and lipid nanodiscs. In contrast, similar substrate binding properties were observed for the small multidrug resistance efflux transporter EmrE in detergent micelles, bicelles and liposomes (19, 20). Interestingly, in a study of membrane helix-helix interactions, significant energetic differences were observed when helices were in bicelles that resembled micelles and bicelles that closely approximated bilayer properties (60). ITC also provides direct means to monitor reconstitution of detergent-solubilized proteins into lipid vesicles, potentially opening avenues for rational optimization of the procedure (62, 63).

### 2.4. Experimental setup

It is typical to load proteins into the reaction cell and ligands into the injection syringe. However, reverse experiments, whereby ligand is in the cell and protein is in the syringe are also possible. Such experiments might be advantageous if the ligand is poorly soluble. Disadvantages of the second setup are that heats associated with protein dilution during injections are typically larger than those observed for small ligands and that higher protein concentrations are needed. Examples of expected titration result are shown in Fig. 1B. In the early part of the experiment, heats associated with ligand-protein interactions are measured, while the later part reflects conditions under which all ligand-binding sites are saturated, and only heats of ligand dilution are measured. Dilution heats are subtracted from the heats of binding during data analysis. It is often assumed that dilution heats are constant throughout the experiment, and hence, an averaged value is subtracted. This assumption is not always true. Therefore, it is recommended to conduct an additional dilution experiment, in which ligand is titrated into the cell containing only buffer. If dilution heats are not constant, then independently obtained values for each injection should be used to correct binding data.

When a new protein-ligand system is considered, the expected binding affinity might not be known. Hence, protein and ligand concentrations selected for the first titration experiment may not be optimal and will need to be adjusted to obtain most informative binding isotherms. Simulation routines are provided with the instruments software to facilitate experimental design, and an excellent new simulator is described in the current issue (64).

### 2.5 Instruments

There are two microcalorimetry configurations differing in size of the reaction cell that are used today: small- (190 – 200 μL) and large- (1.0 – 1.4 mL) cell instruments. Corresponding injection syringes have volumes of 40 – 50 μL and 100 – 300 μL. The small-cell instruments provide higher sensitivity with the minimal detectable heats of ~0.05 μJ compared to ~0.1 μJ of the large-cell instruments. Hence, they reduce sample consumption somewhat. Nevertheless, it is not clear whether small-cell instruments are always preferred. In general, analysis of weaker binding that requires high protein concentrations would usually benefit from small-cell configuration. Conversely, high-affinity interactions requiring dilute protein solutions are likely best analyzed using large-cell calorimeters. Both small- and large-cell instruments allow experiments in a temperature range from 2 to 80 °C.

There are two major producers of ITC instruments: *MicroCal* (now part of *Malvern*) and *TA Instruments*. Table 1 compares two equivalent small-cell instruments. The comparison is not meant to provide exhaustive technical information, but rather to highlight similarities and differences between instruments that are of immediate practical importance. In our experience, *MicroCal* and *TA* instruments provide comparable data quality. An automated *MicroCal* instrument (automated *TA* instrument is under final stages of development) allows increased throughput. This capability facilitates medium throughput analysis of ligands with similar overall properties or other systematic studies where larger volumes of data are required and protein availability is not a limiting factor.

In general, one of the routine challenges in running ITC experiments is maintaining clean sample cell as contaminations result in increased noise and unstable baselines. With membrane proteins, this problem is in part augmented by the use of detergents, in which proteins are solubilized. In our experience, a less thorough cleaning than is recommended by the manufacturers, limited to extensive washes with distilled water after each titration, is preferred in this case. On the other hand, membrane proteins with limited stability may present an additional challenge as they may precipitate during titrations requiring more extensive cleaning.

## 3. Theory

The theory behind ITC experiments has been extensively and thoroughly reviewed (1–4). Here we summarize it only briefly. During an experiment, the power delivered to the reaction cell to maintain its temperature identical to that of the reference cell (typically filled with water) is recorded. When heat is either absorbed or released following ligand injections, amount of the delivered power increases or decreases, respectively, resulting in deflections from baseline. Power delivered to the cell returns to background once temperatures of the reaction and reference cells equalize, producing peaks (Fig. 1). For a fast binding reaction, the response time of the instrument (Table 1) determine the widths of the peaks. However, if binding process is slow compared to the response time, the width of the peaks will also reflect the time required to reach binding equilibrium, providing kinetic information in some cases (65). Notably for slow binding, the time between injections may need to be increased to allow the signal to return to baseline.

Reaction heats are obtained by integrating the power peaks and are typically plotted as functions of molar ratio of the ligand to the protein in the cell (Fig. 1C). The size of these heats depends on the amount of ligand bound after each injection and on the enthalpy of binding

(Eq. 6) $$q_{i}=v\times \Delta H\times \Delta L_{Bi}$$

where *q_i_* is the heat of the *i*-th injection, *v* is the volume of the cell and Δ*L_Bi_* is the concentration increase of the bound ligand following injection. Functional form of Δ*L_Bi_* depends on the mechanism of binding. For the simplest case where a protein contains *n* independent identical binding sites it is determined by Wiseman isotherm (39):

(Eq. 7) $$\frac{{dL}_{B}}{{dL}_{T}}=\frac{1}{2}+\frac{1-\Phi -1/c}{2\times {\left({\left(1+\Phi +1/c\right)}^{2}-4\Phi \right)}^{1/2}},$$

where *Φ* is the degree of titration, defined as *Φ* = *L_T_*/*nM_T_*; *L_T_* is the total concentration of the added ligand; and *M_T_* is the total concentration of the protein. Parameter *c* = *nM_T_*/*K_D_* is described above in **Section 1.2.**

Parameter *n* is strictly speaking not the binding stoichiometry, but rather a ligand to protein molar ratio at which saturation occurs. The value of *n* may be less than 1 if the protein investigated is a multimer binding a single ligand. In this case, *n*, indeed, reflects a lower than 1 stoichiometry of binding. However, *n* value of less than unity may also reflect incorrectly determined protein concentration and presence of contaminants or inactive protein in the sample. We typically observe *n* values between 0.8 and 1 for freshly prepared stable proteins, but *n* often decreases upon protein storage, reflecting deteriorating sample quality. Similarly, *n* values significantly larger than 1 (ideally approaching integer values) are indicative of the presence of multiple binding sites. Data analysis programs provided with ITC instruments incorporate the above fitting equation along with several other models (including multiple non-identical binding sites, sequential binding and simple cooperative models). Many more elaborate binding mechanisms have been implemented in a publically available SEDPHAT program (http://www.analyticalultracentrifugation.com/sedphat/sedphat.htm) (45); others may require custom fitting procedures (66).

## 4. Results and data interpretation

Many membrane protein-ligand interactions has been probed by ITC (5). Most typically, ITC is used to establish functionality of purified proteins, often in support of structural studies. The method has also been applied to probe substrate specificity of transporters and channels and to determine substrate binding stoichiometry. In two studies on membrane transporters (12, 15), ITC was used to examine the thermodynamic linkage between ion and substrate binding to ion-driven transporters. We discuss these studies in detail here (**Sections 4.1 and 4.2**). We also discuss measurements of binding Δ*C_p_* (**Section 4.3**), an underappreciated parameter, which informs on the complexity of the underlying binding mechanism (15).

### 4.1 Cooperative binding of sodium (Na^+^) ions and aspartate to Na^+^/aspartate symporter Glt_Ph_

**Fig. 2:**
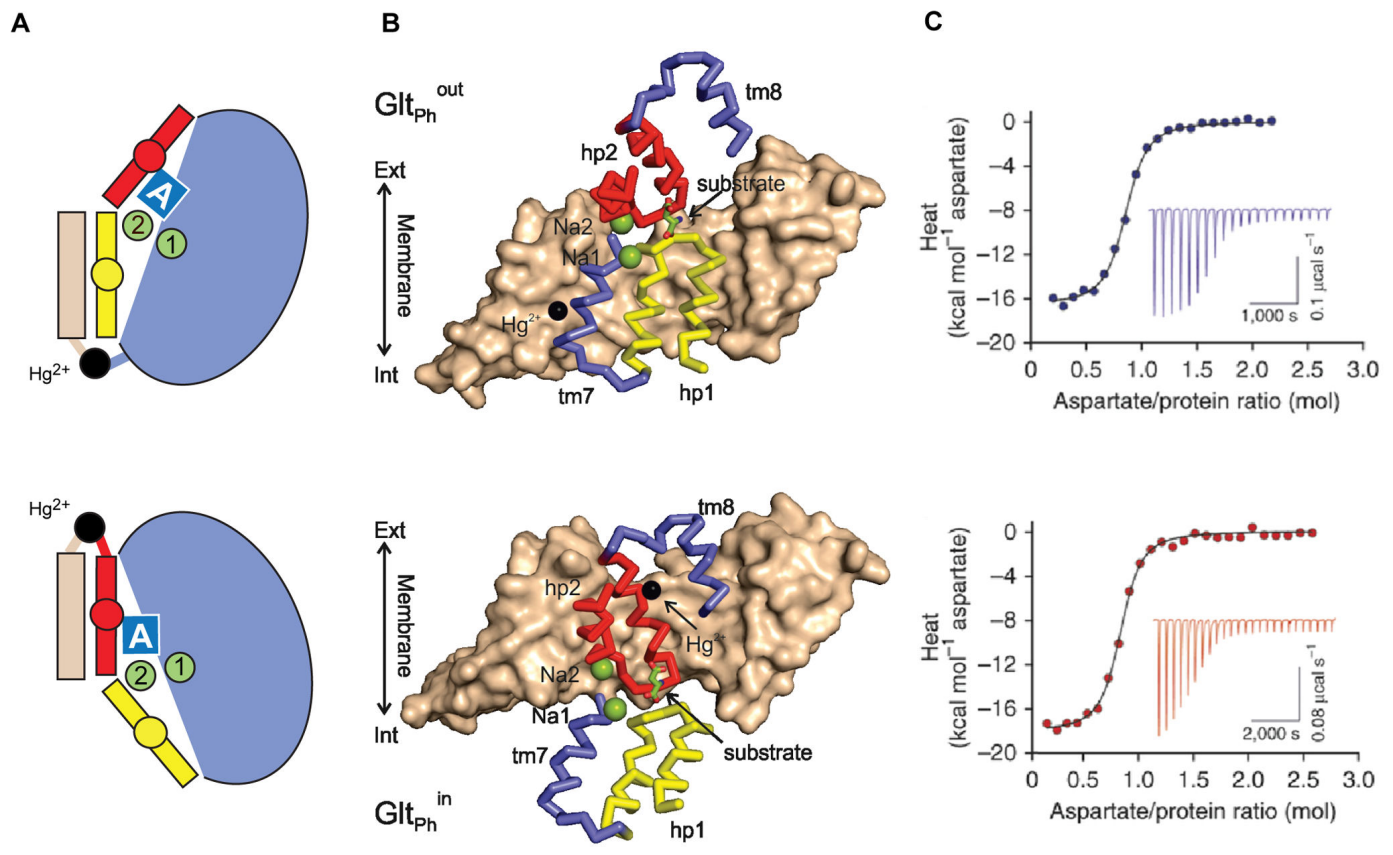
Na^+^-coupled ligand binding to Glt_Ph_ in outward- and inward-facing states (**A**) Simplified cartoon model of single cross-linked Glt_Ph_ protomer in outward- (top) and inward-facing states (bottom). Trimerization scaffold domain is colored wheat; transport domain is blue with gating hairpins (HP1 and HP2) colored yellow and red, respectively. Substrate is represented as a blue box and ions are show as green filled circles. (**B**) Single Glt_Ph_ protomers constrained in outward- (top) inward-facing (bottom) states. Trimerization scaffold domains are shown^•^ in surface representation and interfacial regions of the transport domains are shown as ribbons, with the remainder of the transport domain omitted for clarity. Color scheme is as in (**A**). Bound substrate is shown as sticks and ions as spheres with Na^+^ colored green and cross-linking Hg^2+^ black. (**C**) Examples of ITC binding isotherms for outward- (top) and inward-facing (bottom) states. Integrated heats are shown with baseline-subtracted inverted power data in the insets. Solid lines through the data are fits to the independent binding site model with *K_D_* of 220 and 210 nM, respectively; *n* value of 0.8 for both; and Δ*H* of −16.4 and −17.8 kcal M^−1^, respectively.

Glt_Ph_ is an archaeal homologue of mammalian glutamate transporters responsible for clearance of the neurotransmitter from the synaptic cleft following glutamatergic neurotransmission (14, 67, 68). Functional experiments established that Glt_Ph_ co-transports (symports) each aspartate molecular together with 3 Na^+^ ions. As ions move down their electrochemical gradient from the extracellular solution into the cytoplasm, they provide energy to pump the amino acid against steep concentration gradient (69). In that regard, Glt_Ph_ closely mimics mammalian glutamate transporters, which also symport their substrates (glutamate or aspartate) together with three Na^+^ ions (70). These transport proteins function by alternating access mechanism (71–73): they isomerize between outward- and inward-facing states, in which substrates and ions have access to their binding sites from the opposite sides of the membrane. In Glt_Ph_ and mammalian glutamate transporters, a peripheral transport domain, harboring substrate and ion binding sites, moves ~15 Å across the membrane from an outward- to an inward-facing position within the scaffold of the central trimerization domain (Fig. 2A) (14, 74). The transport cycle of Glt_Ph_ consists of four key steps, all of which are reversible: an empty outward-facing transporter binds substrate and ions from the extracellular space; isomerizes into the inward facing state; releases the solutes into the cytoplasm; and returns into the outward facing state empty.

The energetic coupling of the amino acid uptake to symport of Na^+^ ions requires that neither ions nor substrates be translocated alone. This requirement would be fulfilled if at least one of the following two conditions applied. First, Na^+^ and substrate could bind to the transporter with high positive cooperativity so that they bind poorly in the absence of the symported partner. Second, binding of both solutes could be needed to allow the translocation step to take place. To distinguish between these possibilities, we used ITC to measure aspartate binding to Glt_Ph_ as a function of Na^+^ concentration in the context of the detergent-solubilized protein (15). To prevent Glt_Ph_ from spontaneously sampling outward- and inward-facing conformations, as it typically does (75–78), we introduced intra-molecular cross-links to constrain the transporter in either one of these states (Fig. 2A, B). In so doing, we focused exclusively on binding reactions taking place on the extracellular or cytoplasmic sides of the membrane.

Although Glt_Ph_ assembles as a homotrimer, binding isotherms (Fig. 2C) are consistent with identical independent binding sites with *n* of ~1 per protomer. These results are consistent with previous studies showing that protomers in the trimer function independently. We further found that in the presence of 10 mM Na^+^, aspartate affinities for outward- and inward-facing states were nearly identical and binding enthalpies were similar (Fig. 2C). Consistently, crystallographic studies showed that the transport domain, including substrate-binding site, has identical structure in outward- and inward-facing states both when bound to ligands and when free (apo) (74, 79, 80). However, despite apparent similarities of structure and Δ*G°* and Δ*H* values, the binding reactions are likely mechanistically quite distinct. The differences manifest in much slower binding kinetics (not shown) and much larger binding Δ*C_p_* (discussed in **Section 4.3**) observed in the inward-facing states.

**Fig. 3:**
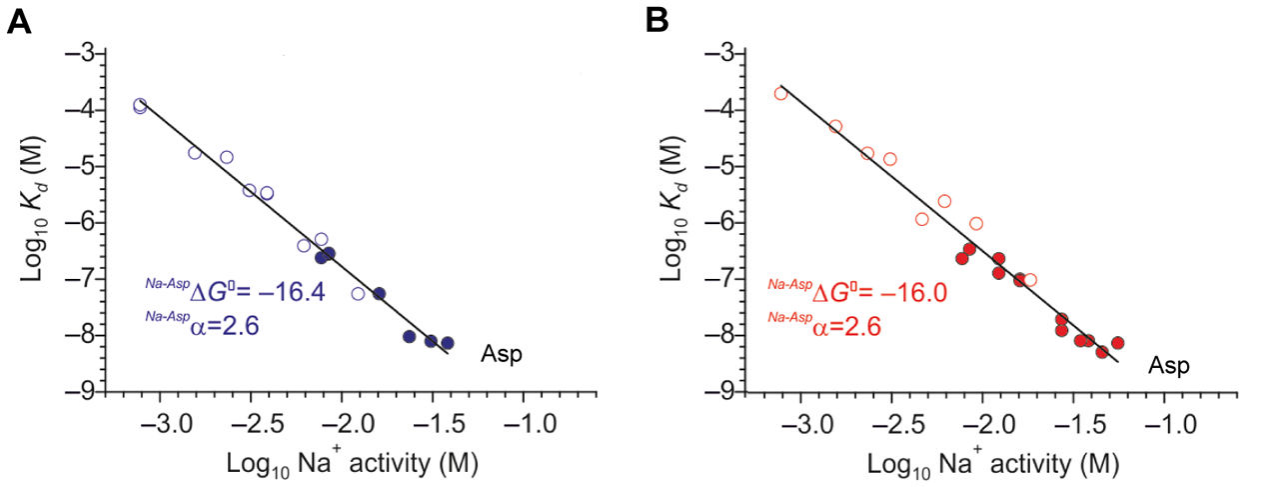
Coupled Na^+^ and aspartate binding ogarithmic plots of the apparent *K_D_*-s for aspartate as functions of Na^+^ activities. Ligand *K_D_*-s for outward- (top) and inward-facing states (bottom) were measured by ITC (filled symbol) and fluorescence-based assay (open symbol). The slopes (a) of the linear fits to the data (solid lines) correspond to the) of to apparent number of Na^+^ ions coupled to ligand binding. Standard free energies of coupled Na^+^ and aspartate binding obtained by extrapolating data to standard conditions of 1 M Na^+^ activity (Δ*G°*, kcal/mol) are shown in the panels. Panels **A** and **B** were adapted and modified from Reyes, *et al*. (15).

Our key finding was that aspartate affinity increased steeply with increasing Na^+^ concentration in both outward- and inward-facing constrained transporters (Fig. 3). To cover the lower range of Na^+^ concentrations, where the affinity for aspartate is too weak to measure by ITC, we augmented calorimetric experiments with fluorescence-based binding assays. To estimate an apparent number of Na^+^ ions (*α*), which bind cooperatively with aspartate, we used the following derivation. The combined dissociation constant *K_D_* for binding of aspartate and Na^+^ is:

(Eq. 8) $$K_{D}=\frac{[M_{F}][\mathrm{Asp}]{\left(a_{{Na}^{+}}\right)}^{\alpha }}{\left[M_{B}\right]},$$

where [M_B_] and [M_F_] are concentrations of the protein bound to aspartate and ions, and of the free protein, respectively; and *a_Na+_* is Na^+^ activity. Assuming that Na^+^ concentration is kept constant during aspartate titration, [M_B_] is equal to [M_F_] when [Asp] = *K_D,app_*. Therefore,

(Eq. 9) $$K_{D,\mathrm{app}}=\frac{K_{D}}{{\left(a_{{Na}^{+}}\right)}^{\alpha }}$$

or, when written in the logarithmic form:

(Eq. 10) $$\log\;(K_{D,\mathrm{app}})=\log\;(K_{D})-\alpha \log\;(a_{Na+}),$$

Implicit in Eq. 10 is the requirement that *K_D,app_*, *K_D_* and *a_Na+_* are normalized by the same unit of concentration, typically 1 M, to render arguments of all logarithms dimensionless. When measured *K_D,app_* for Glt_Ph_ were plotted as a function of Na^+^ activity on a *log*-*log* graph, they fell onto a straight line with the slope, corresponding to *α* (Fig. 3). We found that both in the outwardly- and inwardly-constrained transporter, this slope was ~2.7, suggesting that binding of at least three Na^+^ ions was coupled to binding of one molecule of aspartate. Assuming that only three Na^+^ ions bind to the transporter, as suggested by uptake stoichiometry experiments, all of them bind cooperatively with aspartate. Therefore, in the range of Na^+^ concentrations tested (between 1 and ~100 mM), the ions do not bind appreciably to the transporter in the absence of aspartate. Similarly, aspartate binding to the transporter in the absence of Na^+^ ions is too weak to detect. Notably, Na^+^ ions do bind to the transporter weakly with *K_D_*-s between 100 and 250 mM. Therefore, the observed dependence of aspartate *K_D,app_* is expected to become less steep at higher Na^+^ activities that approach and surpass the ion K_D_-s, reflecting saturation of ion-binding sites.

In consequent crystallographic studies, we used very high Na^+^ concentrations (400 mM) and a Glt_Ph_ mutant with reduced substrate affinity to visualize Na^+^-only bound state (80). Collectively, this and earlier crystal structures showed that transport domain has a compact closed structure in the absence of ligands and when it is bound to both aspartate and Na^+^ ions. In contrast, when it is bound only to Na^+^ ions, its extracellular gate remains open and likely interferes with domain translocation. Thus, both potential mechanisms contribute to enforcing solute symport. However, thermodynamically coupled binding of all three Na^+^ ions and aspartate is likely dominant. Furthermore, Na^+^ ions tightly control substrate affinity of the transporter on the extracellular and in intracellular sides of the membrane: a 10-fold difference in Na^+^ concentration leads to a nearly 1000-fold difference in aspartate affinity. Hence, inward flux of the substrate is achieved when concentrations of the substrate and ions are such that they are more likely to bind on the extracellular side and unbind in the cytoplasm.

### 4.2 Synergistic binding of chloride ions (Cl^−^) and protons (H^+^) to Cl^−^ and H^+^ antiporter CLC-ec1

CLC channels and transporters mediate transmembrane transport of anions (81, 82). In humans, they play diverse physiological roles, including stabilization of membrane potential in skeletal muscles, cellular ion homeostasis, and acidification of intracellular compartments (82). The family is split into *bona fide* channels, which mediate passive rapid diffusion of Cl^−^ ions, and Cl^−^/H^+^ antiporters, which catalyze an exchange of 2 Cl^−^ ions for 1 H^+^ (81, 83). CLC-ec1 is an *E. coli* homologue of the mammalian CLCs, the first member of the family for which coupled ion exchange was demonstrated (83–85).

In the simplest alternating access exchange mechanism, it is expected that transporter isomerizes between outward- and inward-facing states only when it is bound to either one of the exchanged solutes. Moreover, it is typically postulated that the two solutes compete for shared or overlapping binding sites, and states with both solutes bound are prohibited. Therefore, in CLC-ec1 one would anticipate that H^+^ and Cl^−^ compete for binding to the transporter, such that when Cl^−^ ions bind, a proton is released. However, a mutation on the putative proton permeation pathway of CLC-ec1 does not impede Cl^−^ permeation, suggesting that pathways for Cl^−^ and H^+^ are partially separated (86). This unusual organization of CLC-ec1 led to a speculation that Cl^−^ ions and protons may bind to the transporter simultaneously and that the exchange mechanism may not be canonical.

To establish the thermodynamic relationship between the ions, Accardi and colleagues examined coupling between Cl^−^ binding to CLC-ec1 and protonation/deprotonation equilibrium of the protein (12). Toward this end, they used an elegant ITC approach, which takes advantage of the fact that whenever a proton is released by the protein it binds to a buffer molecule. Conversely, if a proton binds to the protein, it has to be released by the buffer (1, 87, 88). Therefore, the overall measured enthalpy of binding, Δ*H_tot_,* has two components:

(Eq. 11) $$\Delta H_{\mathrm{tot}}=\Delta H_{\mathrm{prot}}+N\times \Delta H_{\mathrm{buff}}$$

where Δ*H_prot_* is the enthalpy of protein-specific reactions (Cl^−^ binding and H^+^ binding or dissociation), *N* is the number of protons exchanged between protein and buffer, and Δ*H_buff_* is the enthalpy of buffer ionization, which can be determined calorimetrically under specific experimental conditions (89) and are known for common buffers (90). By measuring Δ*H_tot_* in buffers with distinct ionization enthalpies the number of protons that bind to or dissociate from the protein upon ligand binding is determined from the slope of the dependence of Δ*H_tot_* on Δ*H_buff_*. Negative and positive slopes indicate coupled proton dissociation and binding, respectively.

**Fig. 4:**
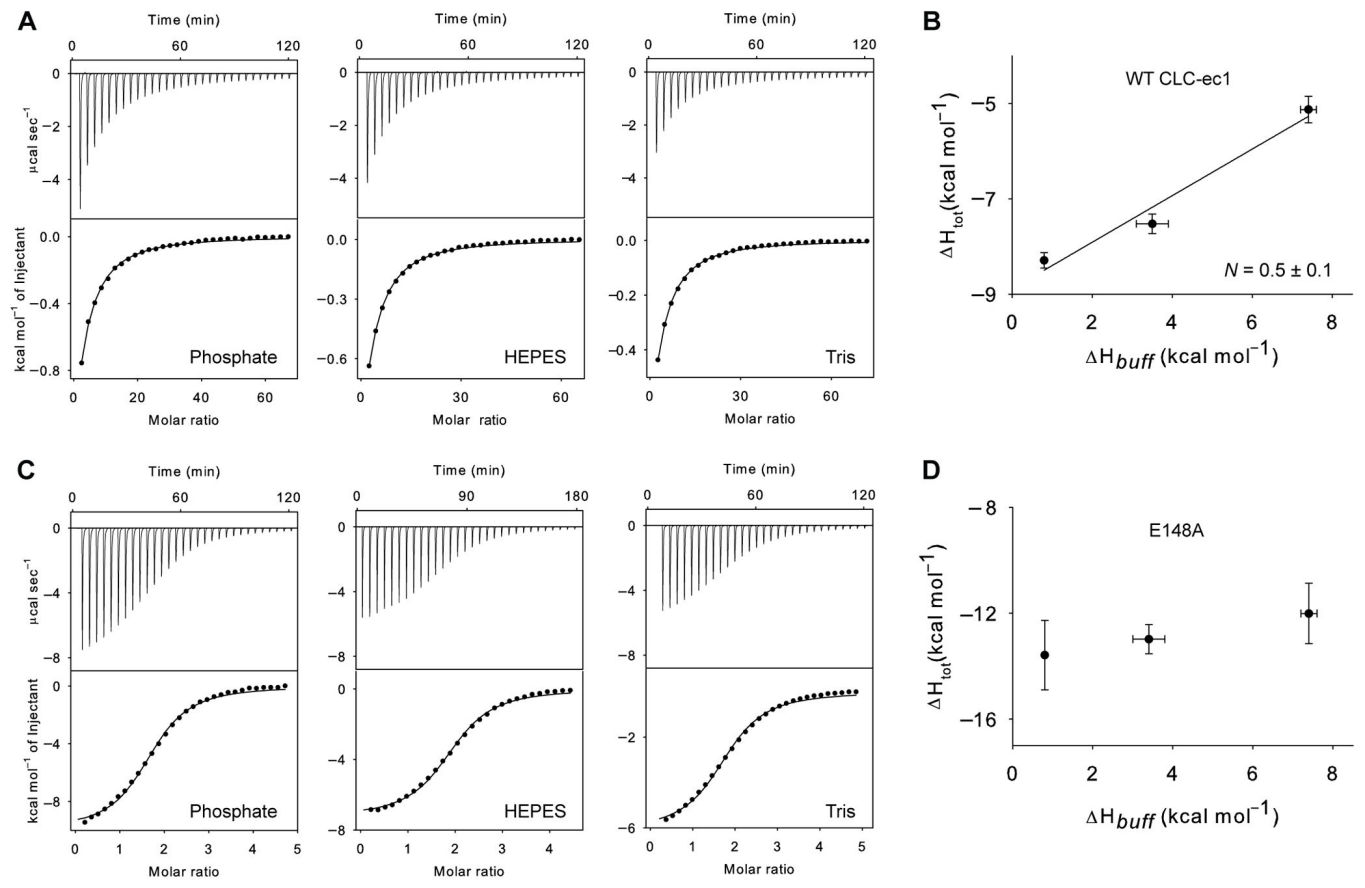
Synergistic binding of Cl^−^ ions and H^+^ to wild type CLC-ec1 (A and B) and to E148A mutant (C and D) ITC data for Cl^−^ binding (**A** and **C**) in phosphate, HEPES, or Tris buffers. In all experiments buffers were supplemented with 5 mM n-decyl-β,D-maltopyranoside Binding heat rates following baseline subtraction (top) and integrated binding heats (bottom) are shown. The solid lines through the data are fits to a single binding site model. The enthalpy of Cl^−^ binding is plotted as a function of buffer ionization enthalpy (**B** and **C**). Error bars are standard errors of the mean. The solid black line in **B** is a linear fit with slope *N* = 0.5 ± 0.1. Figures are adapted and modified from Picollo, *et al.* (12).

**Fig. 5:**
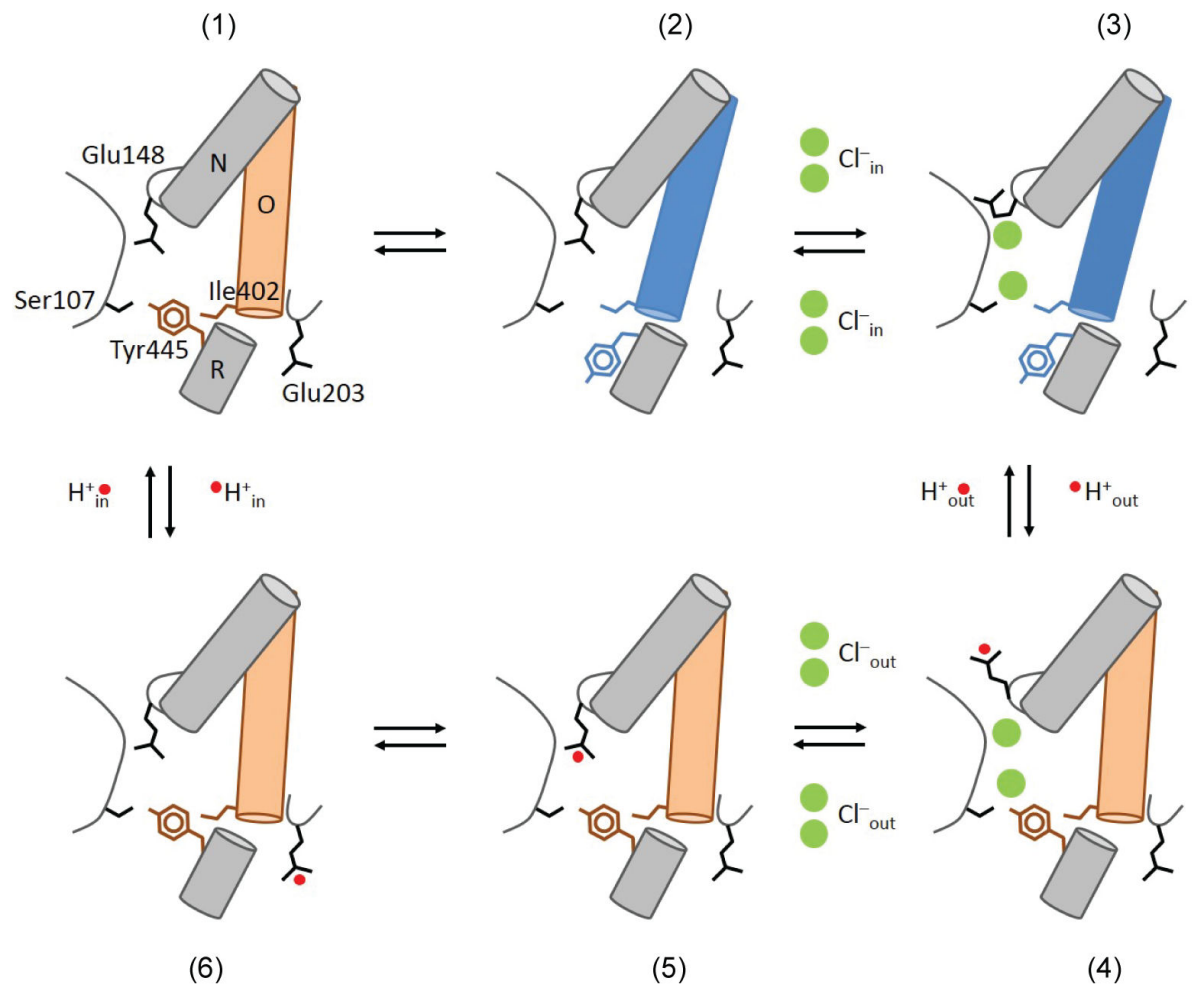
Proposed transport cycle of Cl^−^/H^+^ exchange in CLC transporters The helices and side chains of residues important in ion binding and gating are shown as cylinders and stick, respectively. Blue and wheat colors of helix O emphasize two distinct conformational states. H^+^ (red) and Cl^−^ ions (green) are shown as circles. Details are in the main text. The Figure is adapted from Basilio, *et al.* (92).

Cl^−^ binding to CLC-ec1 is relatively weak with *K_D_*-s around 600–700 μM (Fig. 4A) (12). Therefore, the experimental *c* values were typically < 1 and direct determination of Δ*H_tot_* would have been impossible without a priori knowledge regarding the number of binding sites (as discussed above). Crystallographic studies demonstrated that CLCs have three potential Cl^−^-binding sites, – extracellular (S_ex_), central (S_cen_) and intracellular (S_in_) – defining anion permeation pathway (85, 91). In an earlier study, Accardi’s group coupled ITC experiments with careful mutagenesis perturbing individual binding sites. These experiments established that Cl^−^ binding associated with measurable heats reflected binding to a single site, S_cen_ (11). Binding to S_in_ site appeared to be too weak to detect (estimated *K_D_* over 30 mM). S_ex_ site is occupied in CLCs by a negatively charged E148, which during transport cycle is thought to become protonated and swing out of the protein, freeing the site for Cl^−^ binding (Fig. 5). This notion was supported by the observation that mutation E148A increased affinity of the transporter for Cl^−^ ions and increased the number of bound anions to two (Fig. 4C) (11, 12).

Based on these earlier results, Cl^−^ titration data obtained for CLC-ec1 in buffers with variable ionization enthalpies were fitted to single binding site isotherms (with *n* fixed to 1). Thus obtained Δ*H_tot_* values were then plotted against the ionization enthalpies of buffers determined in independent experiments and fitted to Eq. 11, producing a slope *N* of 0.5 (Fig. 4B). The less than 1 value of *N* may reflect partial occupancy of the proton binding sites prior to Cl^−^ binding or, more likely for CLC-ec1, incomplete protonation upon Cl^−^ binding. While quantitative interpretation of *N* might be difficult considering the potential complexity of the underlying reaction, it is clear that Cl^−^ binding is associated with protein protonation and not H^+^ release.

To establish that the thermodynamically linked protonation reflected binding of the exchanged H^+^ (and not a protonation event at some additional site), Picollo *et al*. examined E148A mutant, lacking the key glutamate involved in H^+^ transport. E148A mutant demonstrated complete elimination of linked protonation (Fig. 4C, and D). In contrast, mutation of another glutamate involved in H^+^ translocation, E203Q, left coupling between Cl^−^ and H^+^ binding unchanged (N = 0.43 ± 0.05). The conclusion form these experiments supported also by computation was that binding reactions of the exchanged Cl^−^ ions and protons in CLC-ec1 were coupled. This was a striking result demonstrating that the exchange mechanism of CLC-ec1 differs substantially from the canonical antiport mechanism, where exchanged solutes compete for a shared binding site. Instead, binding of Cl^−^ ions promoted binding of protons.

This thermodynamic study together with the consequent investigations (92) led to a proposed novel mechanism of exchange (Fig. 5). Briefly, upon spontaneous transition of the occluded empty transporter (state 1) into an inward-open state (state 2), 2 Cl^−^ ions bind from the cytoplasm causing displacement of E148 from the ion permeation pathway (state 3); Cl^−^ binding is also coupled to protonation of E148 and to closure of the intracellular gate (state 4); protonated E148 re-enters ion pathway, displacing Cl^−^ ions into the extracellular solution (state 5); the cycle is completed upon translocation of the H^+^ through its permeation pathway into the cytoplasm (state 6 and 1). The postulated existence of state 4 (prohibited by canonical exchange mechanisms) is based on the detailed observation of synergistic binding between Cl^−^ ions and protons.

By contrast, in a similarly designed study of zinc/H^+^ exchanger Yiip, metal binding was coupled to protein deprotonation, as would be expected for a canonical antiporter (17).

### 4.3 Heat capacity measurements provide insights into the complexity of the binding reaction

Heat capacity is the amount of heat required to increase temperature of a system by 1 K. Equivalently, under conditions of constant pressure, heat capacity *C_p_* is the temperature derivative of mean enthalpy:

(Eq. 12) $$C_{p}={\left(\frac{\partial \langle H\rangle }{\partial T}\right)}_{P}=\frac{\left\{\langle H^{2}\rangle -{\langle H\rangle }^{2}\right\}}{RT^{2}}$$

where < > brackets designate mean value. This equation states that heat capacity is directly proportional to the dispersion of enthalpy between accessible microstates comprising a given state of the protein (33, 93). This relationship makes heat capacity changes the most informative thermodynamic quantity reflecting structural and perhaps dynamic changes in the system. For example, protein folding, protein/protein complex formation as well as some small ligand binding events are associated with large decreases of heat capacity. These had been attributed to the decrease of solvent accessible hydrophobic surface area (33, 93, 94). However, this view has been challenged, and it is probably more accurate to state that any significant increase in the number of weak cooperative interactions leads to the decrease of heat capacity (31, 95). Such increase can be due to the release of water from hydrophobic regions into the bulk, formation of tighter interactions within the protein (rigidification of the structure) and immobilization of water molecules trapped upon ligand binding (31).

Binding of small ligands to pre-existing rigid binding pockets of proteins typically results in small changes of heat capacity, Δ*C_p_*. However, if ligand binding is associated with conformational and dynamic changes in the protein, the values of Δ*C_p_* can be “anomalously” large. An online thermodynamic database (SCORPIO -Structure/Calorimetry of Reported Protein Interactions Online http://www.biochem.ucl.ac.uk/scorpio/scorpio.html) is an excellent resource for examples (96). A particularly striking example is provided by binding of Na^+^ ions to thrombin, which underlies allosteric regulation of the protease. This binding event is associated with a large decrease of heat capacity, which was intriguing because structural changes upon Na^+^ binding were modest (97). While the first hypothesis suggested that an immobilized string of water molecules in the protein core was the source of the large Δ*C_p_*, later work revealed that Na^+^ binding is coupled to rigidification of a flexible autolysis loop (98). Replacing the labile loop with a structurally stable variant increased affinity for Na^+^ ions and overall stability of the protein, and decreased Δ*C_p_* of ion binding. Rigidification of other parts of the protein upon Na^+^ binding was also observed by NMR (99). This example emphasizes that large negative Δ*C_p_*, which cannot be explained by burial of hydrophobic residues, may originate from the reduction of conformational flexibility of the protein. This dynamic feature cannot be observed by crystallography, where a single conformation is selected and stabilized by crystal contacts. Therefore, measurements of binding Δ*C_p_* provide an indirect indication of how extensively the dynamic properties of the system change upon ligand binding.

As an additional benefit, access to Δ*C_p_* allows extrapolation of the binding parameters, including *K_D_,* to temperatures beyond those accessible experimentally (Eq. 2–4). This might be a useful approach when working with proteins such as Glt_Ph_, which originate from thermophilic organisms. While their properties at room temperature are profoundly distinct from mesophilic homologues, they are thought to resemble mesophilic proteins at their corresponding physiologic temperatures in both activity and structural dynamics.

There are two ways to obtain Δ*C_p_* of binding. First, Δ*C_p_* can be determined using van’t Hoff equations from the curvature of log*K_D_* versus 1/T graph. In a more direct way, Δ*C_p_* can be obtained by measuring Δ*H* of binding as a function of temperature (Eq. 3) by ITC. While in the former approach, the second derivative of data is required to obtain Δ*C_p_*, the first derivative is taken in the latter. Thus, this approach is advantageous because a much more narrow range of temperatures needs to be tested and the results are less dependent on small data uncertainties (100, 101). While measurements of binding Δ*C_p_* are common in the analysis of soluble proteins, they have not yet been done routinely for membrane proteins. Procedurally, there should be no significant differences between soluble and membrane proteins.

**Fig. 6:**
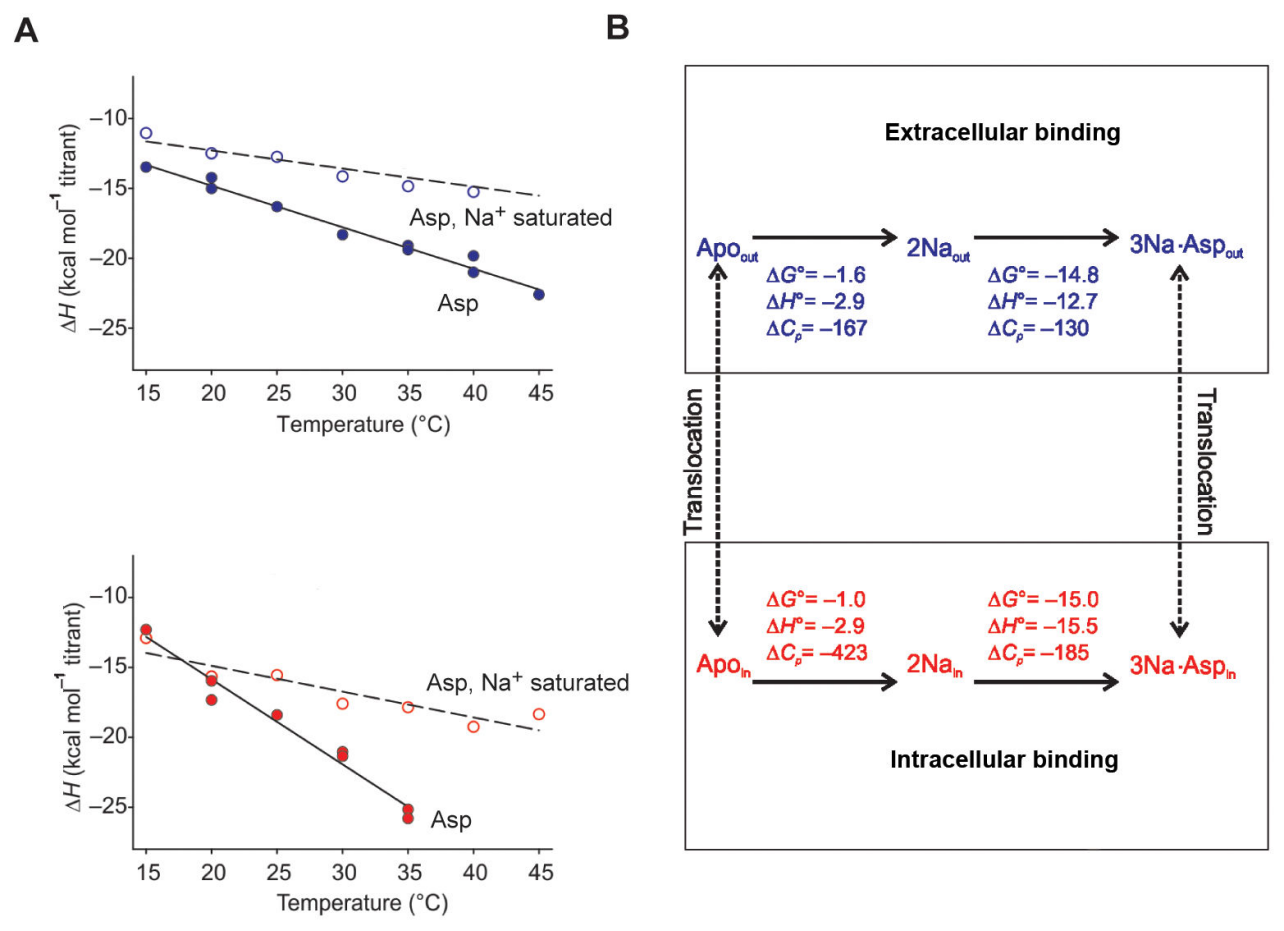
Thermodynamics of ion and substrate binding to Glt_Ph_ (**A**) Determination of binding Δ*C_p_*. Binding enthalpies for aspartate in the presence of 10 mM Na^+^ (solid circles) or 1 M Na^+^ (open circles) are plotted as functions of temperature for outwardly-(top, blue) and inwardly- (bottom, red) constrained Glt_Ph_. The data were fitted to straight lines with slopes corresponding to binding Δ*C_p_* of: −297 and −131 cal mol^−1^ K^−1^, respectively, for the outward-facing transporter and −608 and −184 cal mol^−1^ K^−1^, respectively, for the inward-facing transporter. (**B**) Thermodynamic scheme of the transport cycle of Glt_Ph_. Top and bottom show the thermodynamic schemes of Na^+^ and aspartate binding reactions with corresponding thermodynamic parameters^•^ for the outward- and inward-facing states, respectively. The signs of the parameters correspond to the directions of the reactions indicated by the arrowheads. Broken lines represent conformational changes between the outward- and inward-facing states. The units are kcal mol^−1^ for Δ*H* and Δ*G°* and cal mol^−1^ K^−1^ for Δ*C_p_*. Figure was adapted from Reyes, *et al.* (15).

We measured Δ*C_p_* for binding of Na^+^ ions and aspartate to Glt_Ph_ constrained by crosslinking in either outward- or inward-facing state (15). First, we measured Δ*C_p_* of aspartate binding at relatively low Na^+^ concentration, where binding of Na^+^ and aspartate is cooperative. Δ*H* values determined at temperatures between 15 and 45 °C, revealed linear temperature dependences with slopes of −300 and −600 cal mol^−1^ K^−1^ in the outward- and inward-facing states, respectively (Fig. 6A). These are very large values compared to Δ*C_p_*-s expected for binding of small ligands such as amino acids, which range from ~−10 to −50 cal mol^−1^ K^−1^. For comparison, Δ*C_p_* values expected for folding of a small globular proteins of about 50 residues in length are on the order of −600 cal mol^−1^ K^−1^(102). While from previous and consequent studies (14, 80) we know that Na^+^ and aspartate binding processes are associated with conformational changes of the transport domain, these changes are too modest to explain the large Δ*C_p_*-s observed. The origin of these “anomalously” large Δ*C_p_* values remains unknown. However, it is tempting to suggest (by analogy with Na^+^ binding to thrombin) that ligand binding to Glt_Ph_ leads to rigidification of the protein, perhaps reducing the number of energetically distinct conformational states sampled. This hypothesis, although speculative, is consistent with more recent single-molecule FRET (sm-FRET) imaging experiments (75), suggesting that in apo state, the transporter samples multiple sub-states in both outward- and inward-facing conformations. The sampled conformations appear to differ in strength of interactions between transport and scaffold domains. Substrate binding seemed to greatly favor the states with tight interfacial interactions, perhaps reducing the accessible conformational space.

It is interesting that Δ*C_p_* observed in inward-facing state is much larger than in outward-facing state, although *K_D_*-s and binding enthalpies are not greatly different at 25°C. This disparity further emphasizes that Δ*C_p_*-s measured are dominated not by the complex formation itself (the substrate-binding sites are similar in both states) but by the thermodynamically coupled events involving protein. Furthermore, as the structures of the apo outward- and inward-facing transport domains are also similar, these differences are not due to the differences of the local structure.

To further examine the origin of these large and distinct Δ*C_p_*-s, we determined whether they stemmed from differences in Na^+^ or substrate binding. Binding of Na^+^ is too weak to observe by ITC directly. Instead, we adopted the following experimental protocol. First, aspartate binding parameters were determined in low Na^+^ concentration (as above), where coupled Na^+^ and aspartate binding was observed. Then the experiments were repeated in high sodium concentration (1 M), where Na^+^ binding sites are occupied. Δ*H*-s and Δ*C_p_*-s of Na^+^ binding were then deduced as differences between the first and the second set of values (Fig. 6). Notably, aspartate *K_D_*-s are too tight to measure by ITC under these conditions, but the enthalpies are very well determined. Interestingly, differences in Δ*C_p_*-s of Na^+^ binding in the outward- and inward-facing states mostly accounted for the overall differences, suggesting that the key distinct events take place during ion binding. These results were consistent with our consequent structural studies, which suggested that key transport domain restructuring events were associated with Na^+^ binding (80). These results also suggest that the postulated reduction of conformational flexibility of the apo states is associated with Na^+^ binding.

## 5. Discussion

ITC has been used as a routine tool to measure ligand binding to soluble proteins, and more recently, to membrane proteins. However, its potential in the studies of membrane proteins has not yet been fully realized, and a wealth of mechanistic information might become available with a more extensive usage. At present, ITC is used primarily as a tool to demonstrate protein functionality, i.e. the ability to bind ligands. In this capacity ITC has been extensively applied to probe substrate binding to membrane transporters, where it was also used to establish binding stoichiometry and substrate specificity.

Many membrane proteins - channels, transporters and receptors - are highly dynamic proteins with complex allosteric mechanisms. ITC may provide invaluable information on the energetics of such allosteric relations. Here, for example, we have demonstrated the capacity of ITC to reveal both the number of coupled ions in ion-driven transporters and the mechanism of coupling between them and the substrates. The described approaches can be applied to a diverse range of symporters and antiporters. In a similar manner, ITC can also be applied to studies of ATP-coupled transporters to establish, for example, the thermodynamic linkages between binding of ATP and ADP, and transported substrates.

One class of proteins that has not been extensively studied by ITC is G-protein coupled receptors (GPCRs). In these proteins, allostery plays crucial functional roles, and the energetics of ligand binding might provide very interesting insights. Studies can be designed to investigate coupling between binding of agonists, antagonists and other effectors, and G proteins; or between orthosteric site ligands and allosteric modulators. Similarly in channels, particularly ligand-gated channels, coupling between gating ligands and ions can be probed. Drug optimization strategies based on thermodynamic binding signatures developed for soluble proteins might also be of value here.

Another set of questions that is clearly open for investigations is the effects of lipids on the functional properties of membrane proteins reconstituted into membrane mimetics, such as nanodiscs. These may include simple questions such as whether binding properties of proteins in detergent recapitulate their characteristics in lipid bilayers and more elaborate studies on the effects of specific lipids. Very little is known on this subject. These questions might be particularly interesting in regard to ligand-gated channels and receptors, where ligand binding induces very precise changes in transmembrane domains.

Finally, measurements of heat capacity might provide interesting information on the ligand-dependent dynamic nature of membrane proteins, which is difficult to obtain from other sources. Very large heat capacity and enthalpy changes associated with gating of heat and cold activated transient receptor potential (TRP) family of ion channels have been predicted based on their steep temperature response (103). While these parameters might be difficult to measure directly, they must manifest in the binding thermodynamic signatures of gating ligands that mimic temperature-induced channel opening.

In summary, function-based linkage analyses in membrane proteins, particularly channels and transporters have a very long history. Now, with the advent of membrane protein expression and purification, a new modality to study such linkages by ITC has become available and will likely develop in the coming years.

## Figures and Tables

**Table 1:** comparison of MicroCal and TA small cell instruments

Property	MicroCal iTC200	nanoITC
Sample cell volume	200 μl	190 μl
Sample volume required	280 μl	300 μl
Cell configuration	Coin-shaped	Cylindrical
Cell composition	Hastelloy	Gold
Noise	0.2 ncal/s	0.3 ncal/s
Response time	10 sec	11 sec
Syringe volume	40 μl	50 μl
Syringe filling	Automatic via wash station	Manual
Temperature range	2–80 °C	2–80 °C

## Abbreviations

ITC: isothermal titration calorimetry
CMC: critical micelle concentration
*K_D_*: dissociation constant
Δ*H*: enthalpy change
Δ*S*: entropy change
Δ*G°*: standard free energy change
Δ*C_p_*: heat capacity change
R: gas constant
DMSO: dimethyl sulfoxide
SEC: size exclusion chromatography
Na^+^: sodium
Cl^−^: chloride
H^+^: proton
GPCR: G-protein coupled receptor
TRP: transient receptor potential
